# Tobacco use in the Myeloproliferative neoplasms: symptom burden, patient opinions, and care

**DOI:** 10.1186/s12885-021-08439-7

**Published:** 2021-06-10

**Authors:** Sarah F. Christensen, Robyn M. Scherber, Gina L. Mazza, Amylou C. Dueck, Nana Brochmann, Christen L. Andersen, Hans C. Hasselbalch, Ruben A. Mesa, Holly L. Geyer

**Affiliations:** 1 Department of Hematology, Zealand University Hospital, Roskilde, Vestermarksvej 9, 4000 Roskilde, Denmark; 2 Department of Hematology and Oncology, UT Health San Antonio MD Anderson Cancer Center, 7979 Wurzbach Rd, San Antonio, TX 78229 USA; 3 Hematologic Malignancies, Incyte Corporation, Wilmington, Delaware, USA; 4 Department of Health Sciences Research, Mayo Clinic, 13400 East Shea Boulevard, Scottsdale, AZ 85259 USA; 5 Department of Hematology, University Hospital of Copenhagen at Rigshospitalet, Blegdamsvej 9, 2100 Copenhagen, Denmark; 6 Department of Hematology and Medical Oncology, Mayo Clinic, 13400 East Shea Boulevard, Scottsdale, AZ 85259 USA

**Keywords:** Myeloproliferative neoplasms, Cross-sectional internet-based survey, Tobacco use, Symptom burden, Quality of life

## Abstract

**Background:**

Patients with Philadelphia-negative Myeloproliferative Neoplasms (MPN) suffer from numerous symptoms and decreased quality of life. Smoking is associated with an increased symptom burden in several malignancies. The aim of this study was to analyze the association between smoking and MPN-related symptom burden and explore MPN patients’ opinions on smoking.

**Methods:**

A total of 435 patients with MPN participated in a cross-sectional internet-based survey developed by the Mayo Clinic and the Myeloproliferative Neoplasm Quality of Life Group. Patients reported their demographics, disease characteristics, tobacco use, and opinions on tobacco use. In addition, MPN-related symptoms were reported via the validated 10-item version of the Myeloproliferative Neoplasms Symptom Assessment Form.

**Results:**

Current/former smokers reported worse fatigue (mean severity 5.6 vs. 5.0, *p* = 0.02) and inactivity (mean severity 4.0 vs. 3.4, *p* = 0.03) than never smokers. Moreover, current/former smokers more frequently experienced early satiety (68.5% vs. 58.3%, p = 0.03), inactivity (79.9% vs. 71.1%, *p* = 0.04), and concentration difficulties (82.1% vs. 73.1%, p = 0.04). Although not significant, a higher total symptom burden was observed for current/former smokers (mean 30.4 vs. 27.0, *p* = 0.07). Accordingly, overall quality of life was significantly better among never smokers than current/former smokers (mean 3.5 vs. 3.9, p = 0.03). Only 43.2% of the current/former smokers reported having discussed tobacco use with their physician, and 17.5% did not believe smoking increased the risk of thrombosis.

**Conclusion:**

The current study suggests that smoking may be associated with increased prevalence and severity of MPN symptoms and underscores the need to enhance patient education and address tobacco use in the care of MPN patients.

**Supplementary Information:**

The online version contains supplementary material available at 10.1186/s12885-021-08439-7.

## Background

Tobacco use increases the risk of contracting a broad spectrum of diseases and is one of the leading causes of mortality [1–3]. Cigarette smoke consists of a highly complex aerosol of more than 4000 compounds and induces chronic inflammation with elevated levels of pro-inflammatory cytokines, endothelial dysfunction, activation of leukocytes and platelets, and not least, sustained systemic oxidative stress [4–9]. Interestingly, the Philadelphia-negative chronic myeloproliferative neoplasms (MPN) are characterized by raised levels of several of these cells and inflammatory biomarkers [10, 11]. In recent years, evidence of tobacco use as a contributing factor in the development of MPN has increased [7, 12–15].

MPN are acquired clonal hematologic cancers originating from the hematopoietic stem cell encompassing the three subtypes: essential thrombocythemia (ET), polycythemia vera (PV), and myelofibrosis (MF) [16]. One hypothesis linking tobacco use with MPN includes the possibility that tobacco triggers a low-grade inflammatory state precipitating the development of myeloid mutations and thereby giving rise to MPN with the most critical phenotypic driver mutations being the JAK2V617-, CALR-, and MPL-mutations [11–13, 16]. Patients with MPN are at risk of a wide array of complications, including thrombosis, hemorrhage, bone marrow fibrosis, and progression to acute leukemia. Furthermore, MPN patients often suffer from numerous symptoms such as fatigue, pruritus, bone pain, night sweats, abdominal pain, and reduced quality of life due to splenomegaly, extramedullary hematopoiesis, treatment side effects and also inflammation [17–19]. Recent research indicates an association between individual MPN symptoms and specific markers of inflammation [18]. In line with these findings on inflammation, research in other types of cancer shows that tobacco users have a higher symptom burden than non-tobacco users, a finding that has only been sparsely investigated in patients with MPN [20, 21]. Hence, the aim of this study was to analyze the association between tobacco use and MPN-related symptom burden and, furthermore, explore patient perspectives on tobacco use in an MPN context.

## Methods

### Survey development and collection

The data were obtained in a cross-sectional internet-based survey developed by MPN investigators at the Mayo Clinic and the Myeloproliferative Neoplasm Quality of Life Study group. Patients with a Philadelphia-negative MPN disease were recruited via a posted link on multiple MPN-related web pages, including the *MPN Net, MPN Forum*, *MPN Research Foundation*, and *MPN Voice*. Patients completed the survey between June 22 and July 19, 2018. Data were collected on demographics, disease characteristics (e.g., history of thrombosis, medications), tobacco use, opinions on tobacco use, and MPN-specific symptoms. Participants reported their use of various tobacco products, i.e., smoking, vaping, chewing tobacco, and snuff, as it relates to amount and frequency. Patients were also asked about their use of other substances with abuse potential e.g., screening for alcohol abuse using the validated CAGE questionnaire [22]. Furthermore, participants provided their opinions on smoking risks related to having an MPN disease. The survey was approved by the Institutional Review Board of the Mayo Clinic (IRB No. 18–001151), and informed consent was given by each participant.

### Symptom evaluation and quality of life

MPN-related symptoms were investigated using the validated 10-item version of the Myeloproliferative Neoplasms Symptom Assessment Form (MPN-10) [23]. The MPN-10 asks patients to report the severity of their worst fatigue over the past 24 h; early satiety, abdominal discomfort, inactivity, concentration problems, night sweats, itching, bone pain, and fever over the past week; and unintentional weight loss over the past six months using a 0 (absent) to 10 (worst imaginable) scale. In addition, patients reported their overall quality of life (QoL) using a 0 (as good as it can be) to 10 (as bad as it can be) scale. For patients who completed at least 6 of the MPN-10 items, the total symptom score (TSS) was computed by averaging the available items and multiplying by 10 to achieve a 0-to-100 scale [23]. Finally, the prevalence of each symptom was defined by a score greater than or equal to 1.

### Statistical analysis

Due to a low number of current smokers, the group of *current smokers* (*n* = 20) and the group of *former smokers* (*n* = 161) were combined into a single group of *current/former smokers* (*n* = 181), which was compared with the group of *never smokers* (*n* = 254). When calculating frequencies, patients with a missing response were not included in the denominator. Using SAS 9.4, group differences were evaluated via two-sample *t*-tests for continuous variables and Fisher’s exact test for categorical variables. All statistical hypothesis testing was conducted using a two-sided alternative hypothesis and a nominal significance level of *p* = 0.05.

## Results

### Patient demographics and disease characteristics

Of the 607 MPN patients who clicked the survey link 435 (71.7%) MPN patients (111 males, 324 females) completed the questions concerning smoking history and were eligible for inclusion (Table 1). The MPN patients were of expected age for the disorder (mean 60.7 years, range 26–86), and the distribution of the MPN subtypes were: 119 patients with ET (27.4%), 193 patients with PV (44.4%), and 123 patients with MF (28.3*%)*, of which 58 (47.2%) had primary myelofibrosis (PMF), 41 (33.3%) had post-ET MF, and 24 (19.5%) had post-PV MF. The vast majority were “English speakers” (93.1%), and accordingly, most patients were from the US (66.7%), Australia (11.7%), the UK (6.2%) or Canada (6.2%). The majority of patients received pharmacological MPN treatment, with aspirin (73.1%), hydroxyurea (33.1%) and ruxolitinib (21.8%) being most common. Phlebotomy was currently used in 22.5% of the patients, and 4.8% of the patients required red blood cell transfusions, whereas 1.9% of patients had undergone a splenectomy. In general, current treatment did not significantly differ by smoking status, though current/former smokers were less likely than never smokers to receive aspirin (65.2% vs. 78.7%, *p* < 0.01), but more likely to receive red blood cell transfusions (8.3% vs. 2.4%, p < 0.01). Approximately one quarter (26.0%) of the MPN patients reported a prior thrombosis, while severe bleeding was less common (12.9%).

**Table 1:** MPN Patients’ Demographics by Smoking Status

Demographics	Current/Former (*N* = 181)	Never (*N* = 254)	Total (*N* = 435)	*p*-value
Age (Mean, Range)	61.5 (38.0–86.0)	60.1 (26.0–84.0)	60.7 (26.0–86.0)	0.1879
Gender (*n*, %)				0.0578
Male	55 (30.4%)	56 (22.0%)	111 (25.5%)	
Female	126 (69.6%)	198 (78.0%)	324 (74.5%)	
Country of Residence (*n*, %)				0.1333
US	115 (63.5%)	175 (68.9%)	290 (66.7%)	
Canada	10 (5.5%)	17 (6.7%)	27 (6.2%)	
Australia	30 (16.6%)	21 (8.3%)	51 (11.7%)	
United Kingdom	10 (5.5%)	17 (6.7%)	27 (6.2%)	
Other	16 (8.8%)	24 (9.4%)	40 (9.2%)	
Language (*n*, %)				0.3864
English	166 (91.7%)	239 (94.1%)	405 (93.1%)	
Spanish	3 (1.7%)	1 (0.4%)	4 (0.9%)	
Other	12 (6.6%)	14 (5.5%)	26 (6.0%)	
MPN subtype (*n*, %)				0.0623
ET	44 (24.3%)	75 (29.5%)	119 (27.4%)	
PV	75 (41.4%)	118 (46.5%)	193 (44.4%)	
MF	62 (34.3%)	61 (24.0%)	123 (28.3%)	
MF type (*n*, %)^a^				0.2383
Primary myelofibrosis	34 (54.8%)	24 (39.3%)	58 (47.2%)	
Post-ET myelofibrosis	18 (29.0%)	23 (37.7%)	41 (33.3%)	
Post-PV myelofibrosis	10 (16.1%)	14 (23.0%)	24 (19.5%)	
MPN duration (*n*, %)				0.8305
< ½ Year	6 (3.3%)	13 (5.2%)	19 (4.4%)	
½ - 3 Years	55 (30.4%)	72 (28.6%)	127 (29.3%)	
3–10 Years	65 (35.9%)	92 (36.5%)	157 (36.3%)	
> 10 Years	55 (30.4%)	75 (29.8%)	130 (30.0%)	
Prior bone marrow transplantation (*n*, %)	6 (3.3%)	0 (0.0%)	6 (1.4%)	0.0049
Splenomegaly (*n*, %)	56 (40.3%)	71 (34.3%)	127 (36.7%)	0.4798
Prior splenectomy	6 (3.3%)	2 (0.8%)	8 (1.9%)	0.0725
Prior thrombosis (*n*, %)				0.5062
Blood Clot in Deep Veins of the Leg or Arm	13 (7.3%)	24 (9.6%)	37 (8.7%)	
Blood Clot in Veins of the Head	1 (0.6%)	7 (2.8%)	8 (1.9%)	
Blood Clot in Veins of the Abdomen	7 (3.9%)	13 (5.2%)	20 (4.7%)	
Stroke or Transient Ischemic Attack	15 (8.4%)	21 (8.4%)	36 (8.4%)	
Heart Attack	5 (2.8%)	5 (2.0%)	10 (2.3%)	
Prior hemorrhage (*n*, %)	25 (13.8%)	31 (12.3%)	56 (12.9%)	0.3913
Current treatment of MPN (*n*, %)				
Aspirin	118 (65.2%)	200 (78.7%)	318 (73.1%)	0.0021
Hydroxyurea	56 (30.9%)	88 (34.6%)	144 (33.1%)	0.4696
Interferon	8 (4.4%)	11 (4.3%)	19 (4.4%)	1.0000
Ruxolitinib	41 (22.7%)	54 (21.3%)	95 (21.8%)	0.7258
Anagrelide	9 (5.0%)	6 (2.4%)	15 (3.4%)	0.1833
Phlebotomy requirement	34 (18.8%)	64 (25.2%)	98 (22.5%)	0.1305
RBC transfusion requirement	15 (8.3%)	6 (2.4%)	21 (4.8%)	0.0058
Comorbidity (*n*, %)				
Anemia	101 (56.1%)	101 (40.1%)	202 (46.8%)	0.0004
Heart disease	23 (12.7%)	25 (9.8%)	48 (11.0%)	0.5488
Chronic obstructive pulmonary disorder	11 (6.1%)	(1 (0.4%)	12 (2.8%)	0.0004
Current opioid use for pain management	41 (23.6%)	27 (11.2%)	68 (16.4%)	0.0011

*MPN* Philadelphia-negative chronic myeloproliferative neoplasms, *RBC* Red blood cell

^a^The number of MF patients is used as the denominator: Current/Former Smokers (*N* = 61), Never Smokers (*N* = 62), Total (*N* = 123)

### Smoking characteristics

Of the 435 patients, 254 (58.4%) reported no history of smoking, 161 (37.0%) reported being former smokers, and 20 (4.6%) reported being current smokers. All current smokers (100.0%) and nearly all former smokers (98.7%) reported > 100 cigarettes or vapes in their lifetime (Table 2). The current smokers reported tobacco use “Every day” (73.7%) or “Some days” (26.3%). Current smokers reported smoking premade cigarettes (60.0%), smoking self-rolled cigarettes (35.0%), and/or vaping (5.0%). When separated by the number of cigarettes per day, 36.8% of the current smokers smoked ≥20 cigarettes per day, 31.6% smoked 10–19 cigarettes per day, and 31.6% smoked < 10 cigarettes per day. A few of the former smokers reported smoking, chewing tobacco, and/or vaping “Every day” (3.9%) or “Some days” (3.3%). Vaping was the most common kind of tobacco use among former smokers (Table 2). Most former smokers discontinued smoking > 5 years ago (93.0%), and only 1.2% of the former smokers currently used nicotine replacement. Looking at the total of current and former smokers, 3.3% (*n* = 6) reported vaping, of which 66.7% (4 of the 6 vapers) had switched from smoking to vaping only. All of these patients reported mild (50.0%, *n* = 2) or significant (50.0%, n = 2) improvements in their health following the switch. A higher proportion of current smokers (20.0%) compared with former smokers (5.0%) reported regular exposure to secondhand smoke.

**Table 2:** Tobacco Use Characteristics for Current and Former Smokers

Smoking Characteristics	Current Smokers (*N* = 20)	Former Smokers (*N* = 161)	Total (*N* = 181)
Smoked > 100 Cigarettes or Vapes in Lifetime (*n*, %)	19 (100.0%)	156 (98.7%)	175 (98.9%)
Current Smoke, Chew, or Vape Frequency (*n*, %)			
Not At All	0 (0.0%)	142 (92.8%)	142 (82.6%)
Some Days	5 (26.3%)	5 (3.3%)	10 (5.8%)
Every Day	14 (73.7%)	6 (3.9%)	20 (11.6%)
Type of Tobacco Currently Used (*n*, %)			
Premade Cigarettes	12 (60.0%)	3 (1.9%)	15 (8.3%)
Self-Rolled Cigarettes	7 (35.0%)	1 (0.6%)	8 (4.4%)
Vaping	1 (5.0%)	5 (3.1%)	6 (3.3%)
Chewed Tobacco/Snuff	0 (0.0%)	1 (0.6%)	1 (0.6%)
Nicotine Replacement	1 (5.0%)	2 (1.2%)	3 (1.7%)
Cigarettes Smoked Each Day (*n*, %)			
< 10	6 (31.6%)	–	–
10–19	6 (31.6%)	–	–
≥ 20	7 (36.8%)	–	–
Regular Exposure to Secondhand Smoke (*n*, %)	4 (20.0%)	8 (5.0%)	12 (6.7%)
Time Since Stopped Smoking/Vaping (*n*, %)			
< 6 Months	–	1 (0.7%)	–
6–12 Months	–	3 (2.1%)	–
1–5 Years	–	6 (4.2%)	–
> 5 Years	–	132 (93.0%)	–
Moved from smoking to vaping only (*n*, %)	–	4 (2.5%)	–
Vaping Characteristics			
Times Vaping Each Day (*n*, %)^a^			
< 10	1 (100.0%)	2 (40.0%)	3 (50.0%)
≥ 10	0 (0.0%)	3 (60.0%)	3 (50.0%)
Feel Comfortable Vaping Indoors (*n*, %)^a^			
Yes	1 (100.0%)	3 (60.0%)	4 (66.6%)
No	0 (0.0%)	2 (40.0%)	2 (33.3%)
Health Change After Smoking to Only Vaping (*n*, %)^b^			
Significant Worsening	–	0 (0.0%)	–
Mild Worsening	–	0 (0.0%)	–
No Change	–	0 (0.0%)	–
Mild Improvement	–	2 (50.0%)	–
Significant Improvement	–	2 (50.0%)	–

^a^The number of patients who reported vaping when asked about tobacco use is used as the denominator: Current Smokers (*N* = 1), Former Smokers (*N* = 5), Total (*N* = 6)

^b^The number of patients who moved from smoking to vamping only is used as the denominator: Former Smokers (*N* = 4)

### Smoking and symptom burden

**Fig. 1:**
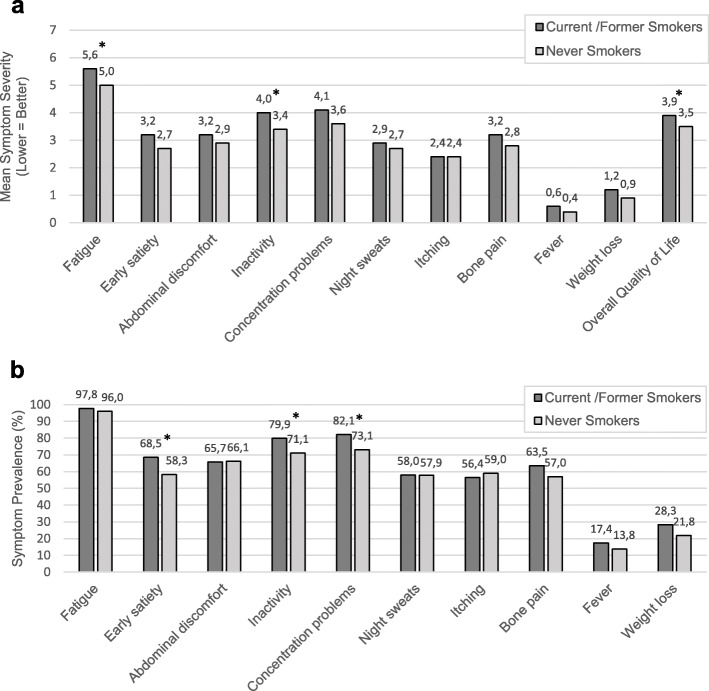
Symptom Severity and Prevalence by Smoking Status. Comparison of mean scores for individual MPN-10 items and quality of life between current/formers smokers and never smokers; **a** Symptom severity was measured on a 0 (absent) to 10 (worst imaginable) scale. Quality of life was measured on a 0 (as good as it can be) to 10 (as bad as it can be) scale; **b** Prevalence was defined as a symptom score greater than or equal to 1. *p-value < 0.05

**Fig. 2:**
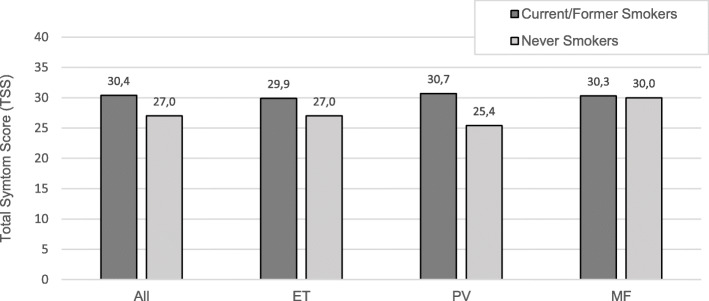
Total Symptom Score (TSS) by Smoking Status and MPN subtype. Comparison of TSS between current/formers smokers and never smokers by MPN subtype. The TSS was calculated for all respondents completing at least six of the 10 MPN-10 items. MPN: Philadelphia-negative chronic myeloproliferative neoplasms; ET: essential thrombocythemia; PV: polycythemia vera; MF: myelofibrosis. No significant mean differences were observed, though *p* = 0.07 for All MPN and *p* = 0.06 for PV

For both current/former smokers and never smokers, fatigue was the most prevalent symptom (97.8 and 96.0%) and also the symptom with the highest mean severity score. Conversely, fever was the least prevalent symptom (17.4 and 13.8%) and had the lowest mean severity score. Current/former smokers reported greater fatigue (mean severity 5.6 vs. 5.0, *p* = 0.02) and inactivity (mean severity 4.0 vs. 3.4, *p* = 0.03) compared with never smokers (Fig. 1.a). Moreover, current/former smokers more often experienced early satiety (68.5% vs. 58.3%, p = 0.03), inactivity (79.9% vs. 71.1%, *p* = 0.04), and concentration difficulties (82.1% vs. 73.1%, p = 0.04) compared with never smokers (Fig. 1.b). Though not significant, a higher total symptom burden was observed for current/former smokers compared with never smokers (TSS mean 30.4 vs. 27.0, *p* = 0.07) (Fig. 2). Finally, and very importantly, never smokers reported better overall QoL compared with current/former smokers (mean 3.5 vs. 3.9, p = 0.03). No significant differences in symptom severity or prevalence were observed for abdominal discomfort, night sweats, itching, fever, or weight loss.

**Fig. 3:**
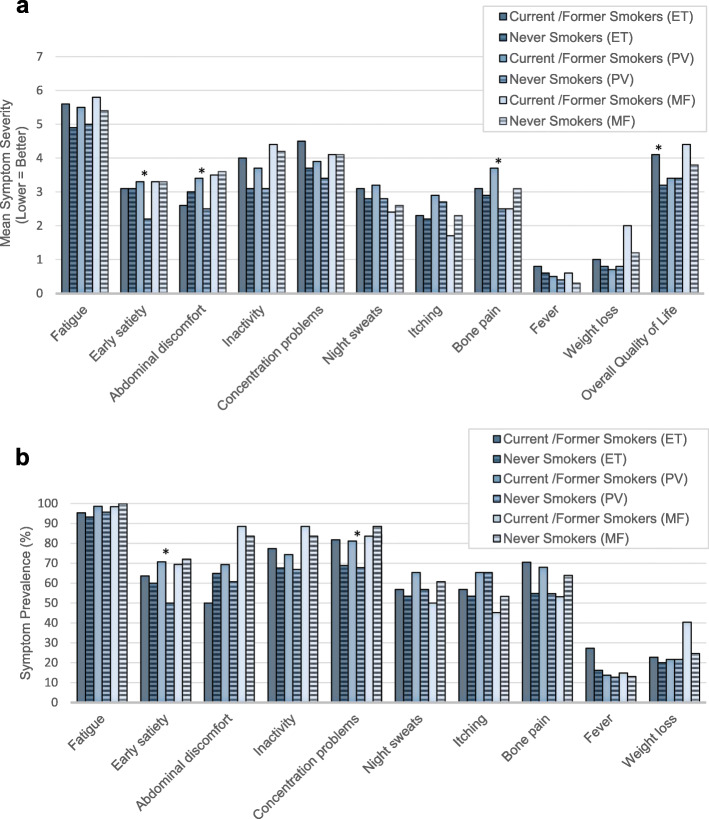
Symptom Severity and Prevalence by Smoking and MPN subtype. Comparison of mean severity (**a**) and prevalence (**b**) for individual MPN-10 items and quality of life between current/formers smokers and never smokers by MPN subtype. Symptom severity was measured on a 0 (absent) to 10 (worst imaginable) scale. Quality of life was measured on a 0 (as good as it can be) to 10 (as bad as it can be) scale. Prevalence was defined as a symptom score greater than or equal to 1. *p-value < 0.05. MPN: Philadelphia-negative chronic myeloproliferative neoplasms; ET: essential thrombocythemia; PV: polycythemia vera; MF: myelofibrosis

When looking within disease subtypes, the same pattern was found for PV and ET patients: ET patients who were never smokers reported greater QoL than the ET patients who were current/former smokers, while PV patients with a current/former tobacco use reported worse early satiety, abdominal discomfort, and bone pain than PV never smokers (Fig. 3a). Furthermore, for PV patients, early satiety and concentration problems were more prevalent among current/former smokers than among never smokers (Fig. 3b). In contrast, no significant differences were observed between MF patients who were current/former smokers versus never smokers.

### Smoking and comorbidity

Current/former smokers more often suffered from chronic obstructive pulmonary disease than never smokers (6.1% vs. 0.4%, *p* < 0.01). Furthermore, anemia around or after MPN diagnosis (56.1% vs. 40.1%, p < 0.01) was more prevalent among current/former smokers with a corresponding higher percentage of current/former smokers reporting a previous or current need for blood transfusions (16.6% vs. 5.1%, *p* < 0.01). In contrast, no significant difference in frequency of heart disease was found (12.7% vs. 9.8%, *p* = 0.55). Only one current/former smoker and one never smoker reported progression to acute leukemia. In terms of mental health history, current/former smokers did not appear to have a higher risk of depression, anxiety, attention-deficit/hyperactivity disorder, schizophrenia, or bipolar disorder, nor did we find a significant difference in history of suicidality. Current/former smokers more often reported heavy alcohol consumption (> 7 drinks per week, 9.6% vs. 4.0%, *p* = 0.03) and were more likely to have a history of alcohol abuse than never smokers (7.2% vs. 0.8%, *p* < 0.01) (Supplemental Table S1). Accordingly, significantly more current/former smokers described being “told to cut back” (10.5% vs. 1.6%, p < 0.01) and that “people had criticized their drinking” (5.0% vs. 1.2%, *p* = 0.03) and further reported “to have felt guilty about drinking” (12.7% vs. 5.9%, *p* = 0.02). Data showed no significant difference in illicit drug abuse besides a higher proportion of current/former smokers than never smokers reporting prior cannabis use (5.0% vs. 0.0%, *p* < 0.01) and use of opioids for pain management (23.6% vs. 11.2%, *p* < 0.01).

### Smoking opinions and care

As shown in Table 3, fewer than half (43.2%) of current/former smokers reported having their physician discuss tobacco use with them. However, this percentage was markedly higher when looking at current smokers separately (85.0%) (Supplemental Table S2). Of the current/former smokers, less than a fourth (23.8%) had tried pharmacological therapy to aid cessation with nicotine replacement being the most common medication used (Table 3). Addressing the effects of tobacco use from an MPN patient perspective, 37.9% of current/former smokers responded “yes” when asked whether smoking increases the risk of developing MPN, while the majority of current/former smokers (82.5%) knew about the increased thrombotic risk. In regards to vaping, only 34.5% of current/former smokers believed that vaping increases the risk of MPN, and 56.6% believed that vaping leads to an increased risk of thrombosis. Approximately one-third of current/former smokers considered vaping safer than smoking for MPN patients.

**Table 3:** Smoking Opinions and Care (current and former smokers)

	Percent responding “Yes” (*N* = 181)
Patients’ opinions regarding the relationship of smoking and MPN	
Smoking Increases Risk of Developing MPN	37.9%
Chewing Tobacco Increases Risk of Developing MPN	36.0%
Vaping Increases Risk of Developing MPN	34.5%
Smoking Increases Risk of Developing Blood Clot	82.5%
Vaping Increases Risk of Developing Blood Clot	56.6%
Vaping Safer Than Smoking for MPN Patients	33.3%
Smoking Cessation Guidance	
Physician Discussed Tobacco Use with You	43.2%
Medications Attempted to Aid Cessation	
Varenicline	3.9%
Bupropion	4.4%
Nicotine Replacements	19.3%

*MPN* Philadelphia-negative Myeloproliferative Neoplasms

## Discussion

With the development of questionnaires specific to MPN symptoms, it has become evident that MPN patients often suffer from a severe symptom burden leading to both affected functionality and reduced quality of life [17, 23, 24]. Targeted treatment with the JAK1/2-inhibitor, ruxolitinib, reduces splenomegaly and constitutional symptoms significantly for some MF and PV patients, but the majority of MPN patients do not benefit from ruxolitinib treatment and discontinuation rates are high due to treatment-related anemia and thrombocytopenia [25, 26]. Hence, the need for interventions alleviating symptom burden is still urgent. However, the multifactorial nature of the disease contributing to the different symptoms complicates matters and stresses the need for a multidirectional approach. A newly published paper on body mass index indicates that underweight and obesity are associated with an increased total symptom score among MPN patients [27]. Accordingly, we hypothesize that smoking represents another, perhaps even more important, contributor to the accumulate symptom burden of MPN. Supporting our hypothesis are two previous MPN studies that found current smokers were more fatigued compared with never smokers [17, 28]. Likewise, recent research demonstrates increased odds of depression and anxiety among MPN patients who currently smoke [29]. However, to our knowledge, the present study is the first to investigate whether total symptom burden (TSS), individual MPN-10 symptoms, and QoL are associated with tobacco use.

In this cross-sectional study measuring symptoms and quality of life with validated questionnaires, we found various interesting results. Overall, our findings indicate that current/former smokers have a higher symptom burden than never smokers, which is in line with research in other malignancies. Studies of patients with prostate cancer have found that smoking has a negative impact on both general and disease-specific symptom burden and QoL. Further support comes from research in both head and neck cancers and lung cancers, showing a negative influence from smoking on symptoms, side effects of cancer treatment, and quality of life [20, 21, 30, 31].

We speculate that the higher symptomatology found among current/former smokers diagnosed with MPN is a result of an accumulated level of chronic inflammation. In healthy, non-smoking individuals, the balance between pro-inflammatory and anti-inflammatory responses is carefully regulated. Conversely, as described comprehensively in two previous published reviews [9, 18], dysregulation of this balance is a hallmark feature of MPN with the Janus kinase cascade playing a key role in the signaling of inflammatory cytokines. The JAK2V617F mutation induces constitutive activation of the Janus kinase generating a measurable elevation of pro-inflammatory cytokines in MPN patients [9, 10, 18, 27, 32, 33]. Likewise, smoking triggers systemic inflammation by releasing and activating acute phase proteins and pro-inflammatory cytokines; thus an additive effect of MPN and smoking seems plausible as many of the elevated inflammatory markers (CRP, IL-1β, IL-6, IL-8, TNFα, GM-CSF and MCP-1) are overlapping [4, 5, 8–10, 18, 27, 34].

In line with evidence of a range of MPN symptoms being associated with specific markers of inflammation, our results show that, on average, current/former smokers reported more severe fatigue than never smokers. This finding is consistent with the results of earlier studies on fatigue in MPN patients [17, 28]. Cytokines, particularly TNFα, have been associated with fatigue in other types of cancer and are, as aforementioned, elevated in MPN [18, 35]. Another known contributor to fatigue is anemia. The association between anemia and smoking is complex with opposing mechanisms. Tobacco use is a risk factor for several diseases (e.g., cancers and gastric ulcers) that are associated with anemia, but the low hemoglobin may be counterbalanced by an increase in the formation of red blood cell due to chronic carbon monoxide exposure. However, even though some stabilization can be achieved, tobacco use, in general, has a negative influence on hematopoiesis [36] and accordingly, current/former smokers more often reported having anemia than their never-smoking counterparts. An established reason for anemia in MPN patients is bone marrow fibrosis. Nevertheless, inflammation, particularly from IL1, IL6, and TNF, might be another contributing cause, promoting deregulation of erythropoietin leading to reduced production of red blood cells [37]. Repeatedly, quality of life studies have highlighted fatigue as the most severe of the MPN symptoms and insufficient treatments are available [17, 23, 28]. Hence, the strengthened evidence of smoking as an aggravating factor of fatigue suggests that smoking cessation counseling may be a beneficial therapeutic intervention.

Besides fatigue, this study identified several symptoms associated negatively with tobacco use; on average, current/former smokers reported significantly higher inactivity scores relative to never smokers. Many reasons may account for this including fatigue, and a significantly higher prevalence of anemia and chronic obstructive pulmonary disease (COPD) among current/former smokers. Moreover, previous research has demonstrated associations between sedentary lifestyles and pro-inflammatory states [18, 38]. Furthermore, current/former smokers were more likely to struggle with early satiety and abdominal discomfort than never smokers. Common causes of these symptoms include splenomegaly, which was also more prominent in current/former smokers. Abdominal thrombosis may also account for these symptoms and interestingly, has been previously associated with elevated levels of CRP, an inflammatory marker elevated in both MPN and smokers [8, 39]. Notably, this study did not find any difference in history of thrombosis between current/former smokers and never smokers but did not examine the possibility of microthrombosis as a contributor. A higher proportion of current/former smokers than never smokers in our study experienced concentration problems which is in line with substantial evidence of smoking’s harmful effects to the brain, The role of oxidative stress and pro-inflammatory cytokines as a link between smoking and cognitive impairment is yet to be fully elucidated, however, it is thought-provoking to note that research in hematologic diseases (and the general population) have shown that increases in circulating inflammatory cytokines, including IL-1, TNFα, IL-6, and CRP, are associated with cognitive deficiencies [18, 40].

When stratifying by MPN subtype we found that for PV patients, current/former smokers experienced worse bone pain than never smokers. This is consistent with previous studies showing increased pain among current smokers with cancer [41]. In contrast, neither symptom severity nor symptom prevalence differed significantly between MF current/former smokers and MF never smokers. We speculate that a contributing reason could be that in the group of current/former smokers with MF, six patients reported a history of bone marrow transplantation compared with none of the patients in the group of never smokers with MF. Assuming that transplanted patients, in general, have a lower symptom score, this would contribute to an overall lower average in the current/former smokers group masking a potential difference due to smoking status.

Of particular interest was the finding that, on average, never smokers reported a significantly better overall quality life compared to current/former smokers. Considering QoL by MPN subtype, we found no significant differences according to smoking status for PV and MF patients while the better QoL among never smokers persisted for ET patients. Whether the association of QoL and tobacco use truly differs between the MPN subtypes or this is an incidental finding due to the relatively small number of patients in some of the strata remains to be explored in future studies. Nevertheless, the better overall quality of life among never smokers with MPN is of great importance and substantiates the majority of existing literature; several studies investigating quality of life in the general population report lower QoL among current smokers and furthermore, looking at cancer patients, findings confirm a worse health-related quality of life among smokers [20, 31, 42, 43]. Compared with other studies, the mean difference QoL between never smokers and current/former smokers was quite small. The small mean difference is probably, at least partly, due to the merge of current smokers and former smokers into one group. A Finnish study [42] demonstrates that former smokers report higher QoL than current smokers, approaching that of never smokers. Another contributing reason could be the use of different questionnaires across studies. Many of the studies reporting a larger difference in quality of life by smoking status use a QoL score calculated from different symptom scores, which is in contrast to the MPN-SAF single item with a self-reported rating of overall quality of life, which is influenced by additional non-health-related factors. From a biochemical standpoint, nicotine ingestion results in activation of nicotinic acetylcholine receptors, which can subsequently modulate the functional pathways related to anxiety, depression and stress response [44]. Many patients experience sedating and calming effects from smoking which could possibly serve as a contributor to improved quality of life.

Evidence states that smoking is associated with other substance abuse and alcohol [30]. Accordingly, we found current/former smokers more often reported higher alcohol consumption, at least two alcohol problems, and an increased sense of guilt related to drinking behavior. As it is widely recognized that alcohol is hepatotoxic, neurotoxic and carcinogenic, and furthermore associated with increased symptom burden and poor QoL, our findings underscore the need to comprehensively screen current and former MPN tobacco users for other harmful substance use behaviors [45, 46].

Despite the irrefutable evidence for the multiple harmful effects from tobacco use, the prevalence of current smokers in the US in 2000 was estimated to be as high as 22.2%, while the corresponding estimates for former smokers and never smokers were 24.4 and 53.4%, respectively [1]. Bearing in mind that our findings are of more recent date (2018), it nevertheless represents a striking difference in usage patterns. A plausible explanation of the lower prevalence of current smokers in our study might be that being diagnosed with cancer is a crucial event in a person’s life, giving rise to new motivation to attempt to quit smoking, a theory with conflicting evidence [30, 47]. Comparing our study population with other MPN cohorts, the prevalence of current smoker is similar to the findings in The Fatigue Study (current smokers, 6%) but markedly lower than in the MPNhealthSurvey (current smokers, 18%), likely reflective of regional variations in usage [28, 48].

The National Comprehensive Cancer Network (NCCN) Clinical Practice Guidelines in Oncology recommends *“Smoking cessation should be offered as an integral part of oncology treatment*” and *“Providers should inform patients of the potential benefits of smoking cessation, including improved survival, treatment outcomes, and health-related quality of life”* [49] Thus, an important finding in this study was the rather high percentage of current smokers reporting having discussed tobacco use with a physician, however as 15% of the current smokers do not recall ever having received tobacco cessation counseling from a physician there is still room for improvement. This is especially true given that almost one-fifth of the MPN patients were unaware of the increased thrombotic risk due to smoking. Patients in our study reported nicotine replacement as the most frequent medication attempted to aid cessation. However, e-cigarettes were also reported as a smoking cessation aid and approximately one-third of the current/former smokers stated that they consider vaping safer than smoking. Interestingly, all of the patients (*n* = 4) who had switched from smoking to vaping reported improvements in their health. Although emerging research suggests some medical disorders are less prevalent with vaping than smoking, this is based on limited data [2, 50]. Hence, much remains unknown about thrombotic predisposition and inflammatory state induced through vaping. Even more elusive are the consequences of vaping for MPN patients; no studies have investigated either acute or long-term complications from vaping in an MPN population. However, a recent study in mice indicates that two months of e-cigarette vapor exposure induces suppression of bone marrow hematopoietic stem and progenitor cells [51]. Furthermore, research in human keratinocytes and epithelial lung cells has demonstrated that excessive vaping induces inflammatory responses activating the JAK-STAT pathway and increasing production of pro-inflammatory cytokines and chemokines (e.g., IL-1, IL-8, and TNF-α) [50]. Bearing in mind the significant role of JAK2 and inflammation in the etiology of the MPN disease, future studies of possible adverse effects of vaping in MPN patients are crucial.

Limitations in our study include the self-reported design with a lack of validation of MPN diagnosis, MPN treatment and smoking status. In addition, MPN mutation status was not included in the survey. Moreover, the patients’ reports of historical data may be affected by recall bias. Another limitation is that *current smokers* and *former smokers* were combined due to a low number of current smokers; this might mask a much larger difference between current smokers and never smokers. Moreover, the cross-sectional design restrains us from drawing conclusions on the causality between smoking status and symptom burden. Also, we cannot rule out that the observed negative association of tobacco use and quality of life, at least partially, could be confounded by other differences between current/former smokers and never smokers, e.g., alcohol abuse and comorbidity. COPD and anemia were more prevalent among current/former smokers than among never smokers, which (along with other comorbidities associated with smoking) might contribute to the observed difference in symptom burden and quality of life. Likewise, symptom scores were not adjusted for the potential impact of current MPN treatment. However, no significant differences in frequency of ruxolitinib or hydroxyurea treatment between current/former smokers and never smokers were present. Finally, when interpreting questionnaire data, it is always relevant to consider if the results are above the minimal important difference, i.e., are of clinical relevance [52]. The mean differences, found in the current study, are quite small, which was expected due to the multiple factors contributing to the severity of the MPN symptoms. However, with no treatment eliminating MPN symptoms available, it is vital to take action against all modifiable causes in order to achieve an overall substantial symptom burden relief. Therefore, despite limitations, we consider many of the findings in this study to be of clinical significance, especially as they relate to potential worsening of the pro-inflammatory MPN environment and opportunities to educate MPN patients on smoking-related risks.

## Conclusion

In conclusion, compared with never smokers, MPN patients with current or former tobacco use reported a higher prevalence and worse severity of a number of symptoms, along with a significantly reduced overall quality of life. It can further be concluded that a considerable percentage of MPN patients are unaware of the increased thrombotic risk related to smoking. Moreover, as approximately one-sixth of the current smokers in our study do not recall ever having discussed smoking with their provider, assessment of tobacco use and subsequent cessation counseling does not appear to be routine practice in the care of all MPN patients yet. Hence, significant benefits may be accrued as they relate to improving health-related quality of life, prevention of comorbidities, and potentially survival. Further studies are crucial to validate our findings addressing the limitations of this study.

## Supplementary Information

**Additional file 1: Supplemental Table S1**. Alcohol, Use and Experiences by Smoking Status

**Additional file 2: Supplemental Table S2**. Smoking Opinions and Care by Smoking Status.

## Data Availability

The data that support the findings of this study and the data processing program codes are available from the corresponding author upon reasonable request.

## Abbreviations

ET: Essential Thrombocythemia
MF: Myelofibrosis
MPN: Philadelphia-negative Myeloproliferative Neoplasms
MPN-10: 10-item version of the Myeloproliferative Neoplasms Symptom Assessment Form
PV: Polycythemia Vera
QoL: Quality of Life
RBC: Red Blood Cell
TSS: Total Symptom Score
