# Supplementary material for: Tobacco use in the Myeloproliferative neoplasms: symptom burden, patient opinions, and care
Source: BMC Cancer. 2021 Jun 10;21:691. doi: 10.1186/s12885-021-08439-7 (PMC8194237; doi:10.1186/s12885-021-08439-7)
Supplement: Supplementary file 1 — Additional file 1: Supplemental Table S1. Alcohol, Use and Experiences by Smoking Status [file 12885_2021_8439_MOESM1_ESM.pdf]

Table S1. Alcohol, Use and Experiences by Smoking Status

Alcohol use and experiences (*n*, %)	Current/Former (*N*=181)	Never (*N*=254)	Total (*N*=435)	*p*-value
Alcoholic drinks per week				0.0164
Do not drink alcohol	84 (47.2%)	126 (50.6%)	210 (49.2%)	
1-7 standard drinks	77 (43.3%)	113 (45.3%)	190 (44.5%)	
8-14 standard drinks	8 (4.5%)	9 (3.6%)	17 (4.0%)	
> 14 standard drinks	9 (5.1%)	1 (0.4%)	10 (2.3%)	
History of alcohol abuse	13 (7.2%)	2 (0.8%)	15 (3.4%)	0.0006
Alcohol use problems				
Have you ever been told to cut back?	19 (10.5%)	4 (1.6%)	23 (5.3%)	<0.0001
Have people annoyed you by criticizing your drinking?	9 (5.0%)	3 (1.2%)	12 (2.8%)	0.0329
Have you ever felt bad or guilty about your drinking?	23 (12.7%)	15 (5.9%)	38 (8.7%)	0.0158
Have you ever had a drink first thing in the morning to steady your nerves or to get rid of a hangover?	2 (1.1%)	0 (0.0%)	2 (0.5%)	0.1726
Number of alcohol use problems				0.0151
0 or 1	170 (93.9%)	250 (98.4%)	420 (96.6%)	
≥ 2	11 (6.1%)	4 (1.6%)	15 (3.4%)	
