# Supplementary material for: Tobacco use in the Myeloproliferative neoplasms: symptom burden, patient opinions, and care
Source: BMC Cancer. 2021 Jun 10;21:691. doi: 10.1186/s12885-021-08439-7 (PMC8194237; doi:10.1186/s12885-021-08439-7)
Supplement: Supplementary file 2 — Additional file 2: Supplemental Table S2. Smoking Opinions and Care by Smoking Status. [file 12885_2021_8439_MOESM2_ESM.pdf]

Table S2. Smoking Opinions and Care by Smoking Status

Patients' opinions regarding the relationship of smoking and MPN	Patients responding "Yes" (*n*, %)		
	Current smokers (*N*=20)	Former smokers (*N*=161)	Total (*N*=181)
Smoking Increases Risk of Developing MPN	7 (35.0%)	59 (38.3%)	66 (37.9%)
Chewing Tobacco Increases Risk of Developing MPN	8 (40.0%)	55 (35.5%)	63 (36.0%)
Vaping Increases Risk of Developing MPN	6 (33.3%)	53 (34.6%)	59 (34.5%)
Smoking Increases Risk of Developing Blood Clot	18 (90.0%)	128 (81.5%)	146 (82.5%)
Vaping Increases Risk of Developing Blood Clot	12 (60.0%)	86 (56.2%)	98 (56.6%)
Vaping Safer Than Smoking for MPN Patients	3 (15.8%)	54 (35.5%)	57 (33.3%)
Smoking Cessation Guidance			
Physician Discussed Tobacco Use with You	17 (85.0%)	59 (37.8%)	76 (43.2%)
Medications Attempted to Aid Cessation			
Varenicline	3 (15.0%)	4 (2.5%)	7 (3.9%)
Bupropion	4 (20.0%)	4 (2.5%)	8 (4.4%)
Nicotine Replacements	10 (50.0%)	25 (15.5%)	35 (19.3%)

MPN: Philadelphia-negative chronic myeloproliferative neoplasms
